# Graphene-Insulator-Semiconductor Junction for Hybrid Photodetection Modalities

**DOI:** 10.1038/s41598-017-14934-4

**Published:** 2017-11-07

**Authors:** Stephen W. Howell, Isaac Ruiz, Paul S. Davids, Richard K. Harrison, Sean W. Smith, Michael D. Goldflam, Jeffrey B. Martin, Nicholas J. Martinez, Thomas E. Beechem

**Affiliations:** Sandia National Laboratories, Albuquerque, NM 87123 USA

## Abstract

A sensitive optical detector is presented based on a deeply depleted graphene-insulator-semiconducting (D^2^GIS) junction, which offers the possibility of simultaneously leveraging the advantages of both charge integration and localized amplification. Direct read-out and built-in amplification are accomplished *via* photogating of a graphene field-effect transistor (GFET) by carriers generated within a deeply depleted low-doped silicon substrate. Analogous to a depleted metal-oxide-semiconducting junction, photo-generated charge collects in the potential well that forms at the semiconductor/insulator interface and induces charges of opposite polarity within the graphene film modifying its conductivity. This device enables simultaneous photo-induced charge integration with continuous “on detector” readout through use of graphene. The resulting devices exhibit responsivities as high as 2,500 A/W (25,000 S/W) for visible wavelengths and a dynamic range of 30 dB. As both the graphene and device principles are transferrable to arbitrary semiconductor absorbers, D^2^GIS devices offer a high-performance paradigm for imaging across the electromagnetic spectrum.

## Introduction

Owing to its high-mobility and broadband optical absorption, graphene continues to be pursued for photodetection applications spanning the ultraviolet^1^ to terahertz^2^ regimes of the electromagnetic spectrum^3^. Achieving sensitivities requisite for practical applications remains elusive however, due to the implicit difficulty of coupling light into an atomically thin layer. In response, nanoantenna enhancement of graphene absorption has been frequently employed^4,5^. This paradigm capitalizes upon sub-wavelength structuring of resonant layers atop the device to effectively concentrate the light into the active graphene channel. While these structures enhance photoresponse, light absorption within the graphene layer typically remains less than 50% of the total incident field. Harvesting the photo-induced electron-hole pairs (e–h) is also challenging since the recombination length in graphene is most often only ∼1 μm^6,7^. For these reasons, photoconducting graphene detectors typically exhibit sensitivities less than 100 mA/W^3^. While bolometric operation circumvents the necessity of carrier collection, it still relies on absorption within the graphene layer itself^8,9^.

To circumvent these difficulties, graphene can be utilized—in an alternative paradigm—as an atomically-thin, transparent, charge-sensing layer that detects absorption within a thicker adjacent substrate, rather than serving as the light-absorbing medium itself. Specifically, the large transconductance of graphene field-effect transistors (GFET) can be leveraged *via* photogating. In this scheme, photogenerated charge created within a semiconducting absorber is capacitively coupled to the graphene thereby inducing a charge of opposite polarity within the graphene channel, modifying the graphene’s conductance. Alterations in the channel conductance can be sensed by measuring channel current during application of a constant bias voltage, mimicking a photocurrent. Since the source-drain current (I_sd_) of the graphene channel is proportional to product of carrier density and mobility (I_sd_ ∝ µnE), graphene’s large ambipolar mobility (~10^3^–10^5^ cm^2^/Vs)^10^ acts as a built-in photogain (i.e., amplifier) mechanism enhancing the detector response. For this reason, sensitivities of photogated graphene devices are higher than other architectures reaching values in excess 1,000 A/W^11–14^.

Photogated graphene detectors have been reported using a variety of absorbers including quantum dots^11–13^, pyroelectric materials^15,16^, and semiconductors^17,18^. Since the graphene in these devices is not responsible for light absorption but only the sensing of charge, absorber choice determines the spectral response. This device concept is thus equally adept at sensing everything from ionizing radiation^19^ to the infrared^20^. For this same reason, detector speed, sensitivity, and dynamic range are impacted by the absorber as well. Detector speed, for instance, is dictated not only by the speed of charge coursing through the graphene channel, but also the rate at which photogenerated charge within the absorber gates the channel. Similarly, sensitivity is not only determined by the transconductance of the graphene channel, but also by the generation-recombination dynamics within the absorber. Lastly, dynamic range is limited by the amount of light collected by the absorber rather than graphene’s density of states (DOS). Full utility can only be realized when both the absorber and graphene are ideally employed as a single system. Here—unlike previous reports of photogated graphene devices^14,17,19^ —we show that by operating a graphene-insulator junction with the semiconductor in deep-depletion, a sensitive photocharge integrating detector with continuous localized read-out (from the graphene) and high-sensitivity can be created. These capabilities, in turn, open up the possibility of alternative detecting modalities in which the advantages of signal integration and continuous monitoring can be leveraged.

Below, the operating characteristics and principles of a D^2^GIS photodetector are presented. Specifically, individual deeply depleted graphene-oxide-semiconductor (D^2^GOS) photodetectors are realized by transferring graphene atop a low-doped silicon substrate that is coated with a thin oxide. The fabricated devices are analyzed with respect to their photoresponse in the visible. Possessing responsivities exceeding 2,500 A/W, an SNR of ~ 3, and up to 30 dB of dynamic range at room temperature, the device response is explained entirely through a semi-analytical calculation taking into account the basic transfer characteristics of a graphene field-effect transistor combined with depletion and subsequent carrier generation in a low-doped semiconductor.

## Results and Discussion

### D^2^GOS Detector Architecture and Theory of Operation

**Figure 1:**
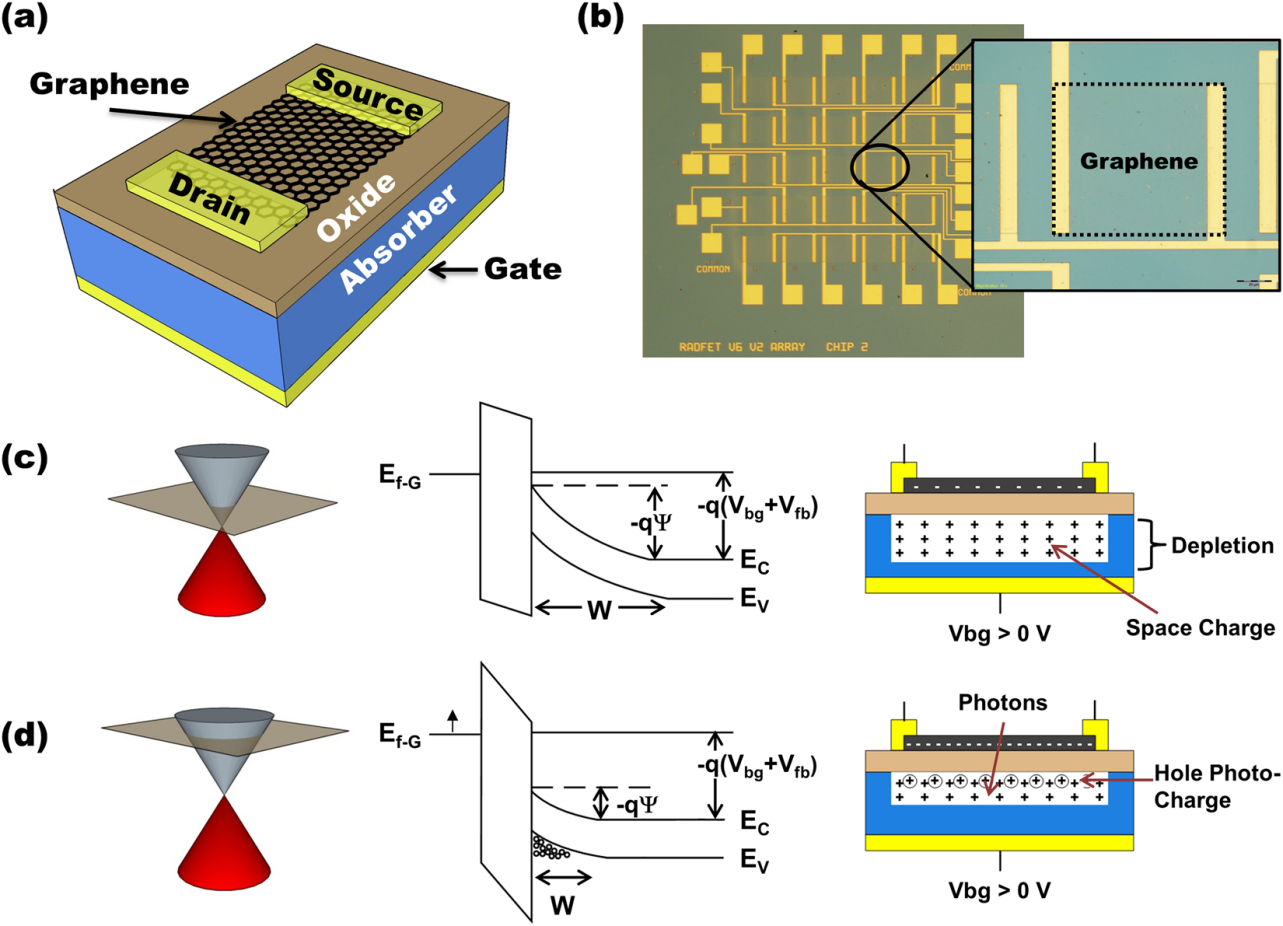
Description of device and operational principles. (**a**) 3D-schematic of the graphene/oxide/semiconductor detector. (**b**) Optical image of several D^2^GOS devices. Inset: Zoom in on an individual 200 µm × 200 µm detector. (**c**) Band diagram when the GOS junction is brought into deep depletion by a momentary voltage pulse applied to the back-gate, where E_f-G_ is the graphene Fermi-level, E_c_ is the conduction band level, E_v_ is the valance band level, and V_bg_ is the back-gate voltage with respect to ground. The graphene is assumed to be lightly n-doped when a V_bg_ > 0 V is applied. (**d**) With illumination, photo-induced holes and dark charge accumulate in the potential well, inducing electrons in the graphene, raising the Fermi-level and increasing the conductivity of the channel.

Figure 1a illustrates the D^2^GOS detector concept. The device consists of a back-gated graphene-oxide-semiconductor (GOS) junction. Graphene channel conductance is measured using source-drain electrodes with constant bias application. To sense photo-generated charge, the semiconductor absorber is brought into deep depletion by rapid application of a back-gate voltage (V_bg_). Figure 1c shows the energy band diagram of the GOS junction, using n-type Si, initially biased into deep depletion with rapid application of V_bg_ > 0 V. Positive V_bg_ forces mobile electrons to diffuse into the bulk Si, exposing the space charge within the depletion zone of width W. The surface potential (ψ_S_) at the silicon/oxide (Si/ox) interface goes negative and the electric field within the depletion region separates photo-generated e-h pairs resulting in the collection of holes at the Si/ox interface.

Since the graphene is capacitively coupled to the underlying Si absorber, the collected holes induce electrons in the graphene channel, modifying the Fermi-level, thus altering the film’s conductivity (Fig. 1d). Modification of the graphene conductivity is detected by measuring changes in the source-drain current with application of a small voltage across the graphene channel. As time increases under continuous illumination, the hole density at the Si/ox interface increases causing the surface potential to decrease (Fig. 1d)^21,22^. This behavior is similar to the integration mechanisms of a classical deeply depleted MOS capacitor with the exception of the top-gate electrode being replaced with a graphene channel that locally senses charge at the Si/ox interface.

### Characterization of D^2^GOS Optical Response, Signal-to-Noise, and Dynamic Range

To demonstrate the device described above, D^2^GOS detectors were fabricated by transferring chemical vapor deposition (CVD) derived graphene onto a lightly doped n-type silicon absorber (N_d_ ~ 10^14^ cm^−3^) capped with 50 nm of HfO_2_ that was deposited by atomic layer deposition (ALD) (see Methods). The doping level of the Si substrates was verified using secondary ion mass spectrometry (SIMS) and capacitance-voltage (CV) measurements (see Supporting Information). Figure 1(b) shows an optical micrograph of several independent D^2^GOS detectors with the inset highlighting an individual 200 × 200 µm device. Detectors exhibiting characteristics similar to those reported below were fabricated on several silicon substrates and all results were repeatable across multiple devices indicating the robustness of this design. While the devices characterized here employed silicon absorbers, the methodology is portable to other suitable absorbers making the concept applicable for detector design throughout the visible and infrared. When selecting alternative absorbers, the interplay between several factors must be considered: 1) Fermi level alignment between the graphene and absorber, 2) voltages required to reach graphene charge neutrality and absorber depletion, 3) formation of low-defect absorber/insulator interface, and 4) carrier lifetime.

**Figure 2:**
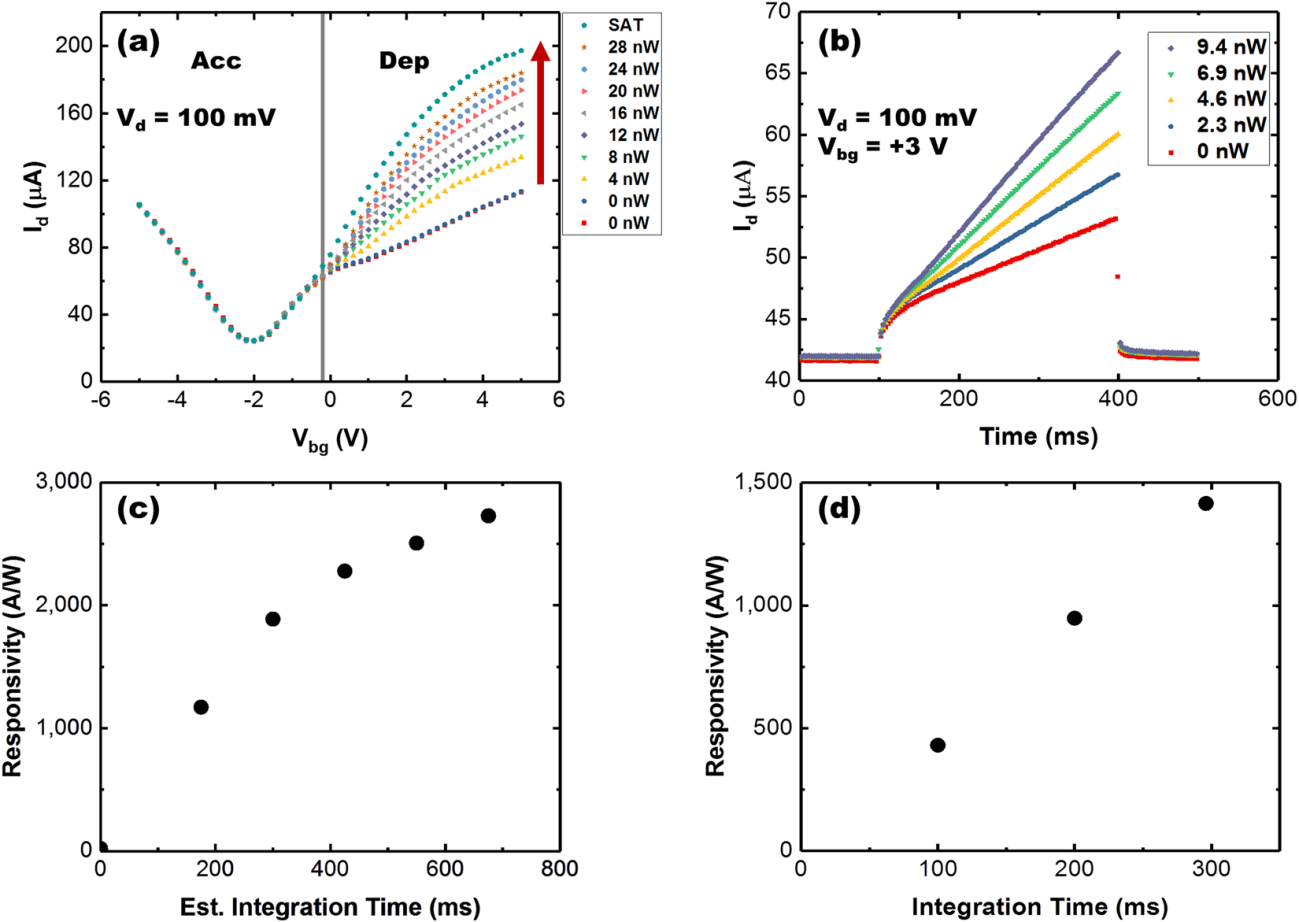
Device response under dark and illuminated conditions as a function of gate voltage. (**a**) I_d_(V_bg_) of a typical D^2^GOS detector under different illumination using a 635 nm laser (red arrow indicates increasing illumination). The vertical line in (**a**) divides the plot into the accumulation (Acc) and depletion (Dep) regions. For these data, the voltage step time (instrument integration time), which is used to deduce the amount of time the potential well has been collecting photogenerated charge for a particular V_bg_ value, was estimated to be ~ 20–25 ms (see SI). (**b**) I_d_ response as a function of time and optical power. A bias of 3 V is applied at a time of 100 ms and turn off at 400 ms. Responsivity as a function of integration time when V_bg_ is (**c**) swept and (**d**) held constant (pulsed).

To characterize the optical response, devices were exposed to 405 nm and 635 nm laser light of varying optical power (P_opt_) in an otherwise optically dark environment (see Methods). For these measurements, the current at the drain electrode (I_d_) was measured during application of 100 mV drain voltage (V_d_) while the source electrode was held at ground. Figure 2a,b show the photoresponse (I_d_) of a D^2^GOS detector acquired during two different modes of operation. Figure 2a displays the photoresponse of a D^2^GOS device during application of a back-gate voltage sweep where the Si is first biased into accumulation and then swept into depletion. Figure 2b, meanwhile, provides the photoresponse as a function of time when the Si absorber is biased into deep depletion by rapid application of a back-gate voltage, which is then kept fixed. Care was taken to ensure that the sweep and acquisition rates used for these measurements were sufficient to bring the Si absorber into deep depletion (see Supporting Information).

We first discuss the device’s behavior in the absence of illumination (0 nW traces shown in Fig. 2a). The graphene charge neutrality point (V_CNP_) is at ~ −2 V, indicating that the graphene is n-type possessing a residual carrier concentration of ~1.3 × 10^12^ cm^−2^ (see Supporting Information). The resulting Dirac curve is similar to that observed for GFETs atop a degenerately-doped semiconductor substrate except for an interesting difference when V_bg_ approaches −0.2 V. Specifically, a distinct reduction in transconductance is observed in this region. This behavior is not observed when a degenerately doped semiconductor is used as the substrate. In contrast, under heavy illumination (saturated trace in Fig. 2a), the reduction in transconductance is absent with the I_d_ response appearing like that of graphene on a heavily doped Si substrate.

These transfer curves can be understood by investigating the changes that occur at the low-doped Si/ox interface with gate bias. For a back-gated nMOS capacitor, the system is biased into accumulation when V_bg_ is less than the flat-band voltage (V_fb_) whereas it is depleted when V_bg_ > V_fb_. CV measurements of Au/HfO_2_/Si capacitors adjacent to the D^2^GOS detectors (see Supporting Information) exhibit a flat-band voltage of near −0.2 V. Thus, owing to the similarity in work function between the Au and graphene^23^, the drastic reduction in GFET transconductance near −0.2 V when unilluminated arises due to the drastic reduction in capacitance commensurate with the flat-band voltage. Simply stated, the GFET is particularly sensitive to the charge environment at the Si/ox interface. Sensing charge at this interface is the basis for light detection and D^2^GOS detector operation.

Consider the difference in device response (I_d_) when the gate biases silicon into accumulation (V_bg_ < −0.2 V) versus depletion (V_bg_ > −0.2 V). When biased into accumulation, mobile electrons in the n-type Si collect at the Si/ox interface. The junction behaves like a parallel plate capacitor, where electron charge present at the Si/ox interface depends on V_bg_. This charge is approximated by Q_Si_ = C_ox_(V_bg_–V_fb_), where C_ox_ is the oxide capacitance and Q_Si_ is the areal charge density at the Si/ox interface. The voltage induced electron density at the Si/ox interface, in turn, is balanced by an equivalent hole density within the graphene channel. The increased hole density alters the conductance of the graphene channel. In accumulation, recombination rates in the Si increase with the carrier density. As a result, virtually no photoresponse exists when the device operates in accumulation (see V_bg_ < −0.2 V in Fig. 2a).

When depleted, mobile electrons are swept away from the Si/ox interface and into the bulk silicon, leaving behind a space charge region. The electric field associated with the space charge region separates photogenerated e-h pairs with near perfect efficiency^21^. Holes therefore collect at Si/ox interface when the device is illuminated. Electrons are created within the graphene channel to balance this charge causing an increase in current proportional to the product of this charge and the graphene mobility. This change in current is the photoresponse as is shown in Fig. 2(a). Similar results were observed for multiple devices illuminated with both 405 nm and 635 nm light (see Supporting Information).

Due to the rapid carrier recombination (~2 ps^24^) in graphene and the size of our devices (hundreds of microns), direct photoconduction within the graphene channel is presumed negligible. This claim is supported by the lack of any measureable change in the drain current during accumulation when exposed to different optical powers (Fig. 2a).

As a depleted semiconductor collecting minority carriers at a semiconductor/oxide interface, the silicon substrate functions much like it does in a traditional deeply depleted MOS capacitor. The D^2^GOS device possesses a distinguishing difference unavailable with a deeply-depleted MOS capacitor, however. Namely, the charge integration process is directly monitored and visualized by sampling the graphene’s conductance as a function of time.

This ability is quantified explicitly by recording the drain current after sudden application of a positive gate voltage that induces depletion within the Si (visualizing the charge integration process). Figure 2b (where V_bg_ = +3 V) shows a typical D^2^GOS detector photoresponse at a range of incident optical powers when rapidly biased (<1 µs) into deep depletion with a point-to-point sample read-out time of 3 ms (see Methods). In all cases, as charge collects during deep depletion, the graphene’s conductivity increases linearly (increasing I_d_) due to an increasing electron charge density created by capacitively coupled hole charge in the potential well. For example, drain current increases linearly with incident power. Therefore, the signal is being integrated even as the detector is being continuously read-out. This dual-capability is a differentiating feature of the D^2^GOS detector. Of note, consecutively collected acquisition loops showed minimal hysteresis (see Supporting Information). Therefore, charge is being integrated at the interface rather than being dictated by traps within the HfO_2_ dielectric.

Since collection times for the illuminated device presented in Fig. 2a,b are known, it is possible to extract time dependent responsivities (see Supporting Information). Figure 2c,d show the measured responsivities extracted from the I_d_(V_bg_,P_opt_) and I_d_(time,P_opt_) data. Since the D^2^GOS device possesses a hybrid modality, charge integration with an electrical current readout, an integration time dependent responsivity is a useful metric for quantifying detector performance. While both methods of back-gate biasing give time dependent responsivities that are approximately consistent, we suspect that the slight discrepancy is due to uncertainties associated with estimating voltage sweep timing and the onset of depletion. Due to these uncertainties, the estimated integration times may be slight larger than the reported estimates in Fig. 2c. In contrast, the timing of the pulsed gate method is precisely known and those reported time dependent responsivities are more representative of actual device operation.

Current is transported through the device even when it remains in the dark (see red squares in Fig. 2b). The slope of I_d_ at 0 nW is due to dark charge collecting in the potential well at the Si/ox interface. The sources of this accumulated dark charge derive from exposed positive space-charge and holes generated by thermal and surface generation processes. Functionally, dark charge reduces the capability of the detector by both serving as a source of noise while also filling the well thereby reducing dynamic range. To quantify these non-idealities and the detector performance, the signal to noise ratio (SNR) was calculated by (adopted from refs^25^ and^26^):

1 $${\rm{SNR}}=\frac{{\rm{Sdt}}}{\sqrt{{\rm{Sdt}}+{\rm{Ddt}}+{{\rm{N}}}_{{\rm{Readout}}}^{2}}},\,$$

where S is photo-generated signal per unit time, D is the dark charge signal per unit time, and N_Readout_ is the signal read out noise. To estimate SNR, dark (I_d-Dark_(t)) and illuminated (I_d-Light_(t)) data are used. For these estimates Sdt = I_d-Light_(t)–I_d-Dark_(t) and Ddt = I_d-Dark_(t)–I_d_(0), where I_d_(0) is the offset drain current prior to application of the V_bg_ pulse. Readout noise (${N}_{Readout}^{2}$) derives primarily from thermal, shot, and 1/f (flicker) sources. These were quantified by taking the Fourier transform of the drain current measured in the dark over a 10 s period, subject to a sampling rate of 333 Hz, resulting in a normalized spectral noise density (S_I_/I^2^) spanning from 10^−6^ Hz at low frequency to 10^−9^ at frequencies > 30 Hz. Readout noise at the sampling rate of 333 Hz is inconsequential relative to that arising from dark processes, possessing a magnitude of ~50 nA.

**Figure 3:**
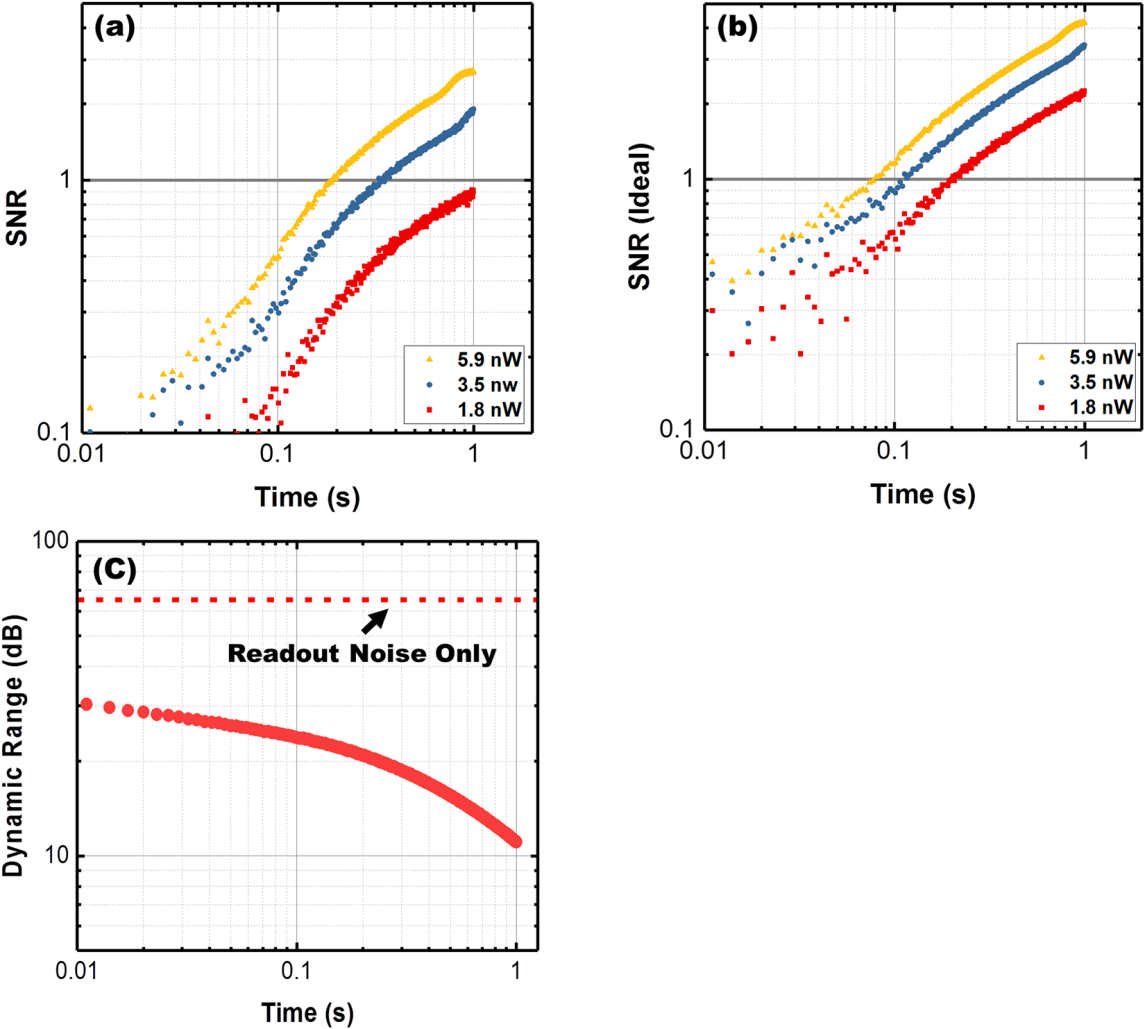
Characteristics of detector performance extracted from I_d_(time) data, in Fig. 2b, under dark and low-level illumination conditions. (**a**) D^2^GOS detector room temperature SNR as a function of collection time. (**b**) An ideal SNR estimate for the case where only the photo-signal is considered (simulating the condition where the dark charge signal is minimized by cooling). (**c**) Dynamic range for different integration times (extracted for I_d_(time) data). These data indicate a dynamic range between 10–30 dB at room temperature.

Figure 3a presents the SNR at room temperature, extracted directly from current traces of an D^2^GOS detector, operating at room temperature as a function of integration time for illumination powers ranging from 1.8–5.9 nW. SNR near 3 is found using modest integration times (t < 1 s) and low illumination levels (<5.9 nW). Importantly, SNR increases with longer integration time. However, due to the graphene-enabled readout of the collecting charge, signal can be constantly monitored at speeds determined only by the rate of graphene photogating. Figure 3b presents an estimate of improvements to SNR through device cooling. These data exclude the dark charge signal, which directly results from thermal effects.

The dynamic range is determined by the charge storage capacity of the potential well, the time-dependent dark charge filling it, and the readout noise level. Well capacity was measured by monitoring the source-drain current to saturation in the dark after a gate bias of 3 V was rapidly applied (see Supporting Information). Saturation times of 8–9 seconds were observed at room temperature correlating to a well capacity of ϕ_tot_ = 0.8 µC/cm^−2^, which is near the predicted capacity of ϕ_max_ = C_ox_V_bg_ = 0.9 µC/cm^−2^ ^21^. Given our device size of 200 × 200 µm^2^, this corresponds to a well capacity of ~2 × 10^9^ and ~2.25 × 10^9^ electrons respectively.

Knowing these characteristics, dynamic range (DR) of the D^2^GOS detector is calculated *via* ^27^

2 $${\rm{DR}}=20{\mathrm{log}}_{10}(\frac{{{\rm{I}}}_{{\rm{d}},{\rm{Sat}}}-{{\rm{I}}}_{{\rm{d}}}(0)}{{{\rm{I}}}_{{\rm{d}},{\rm{Dark}}}({\rm{t}})-{{\rm{I}}}_{{\rm{d}}}(0)+{{\rm{I}}}_{{\rm{Noise}}}}),$$

where I_d,Sat_ is the drain current of a saturated well, I_d_(0) is the drain current just prior to application of the back-gate voltage (t = 0), I_d,Dark_(t) is the time dependent drain current under dark illumination, and I_noise_ is the graphene noise current at the sampling time of 3 ms. As shown in Fig. 3c, the D^2^GOS detector exhibits a room temperature dynamic range of 10–30 dB depending on integration time. Removal of dark processes—as could be attained under Peltier based cooling—results in an estimated DR of ~60 dB as shown by the dotted line of Fig. 3c and an increase of SNR as shown in Fig. 3b. These values are limited by the quality of the Si/HfO_2_ interface rather than the graphene as the dark term dominates Eq. 2. Further optimization of the interface will thus likely provide drastic improvements in DR and SNR.

### Predicting Performance and Prospects

To further understand the underlying physics of the device, a semi-analytical model was developed to predict device performance and assess future potential. The simulation couples an analytical model of a MOS capacitor biased into accumulation or deep depletion^21^ with calculation of current transport through a graphene FET influenced by charge at the oxide interface^28^ (see Supporting Information). The simplistic nature of this model does not capture every detail of the mechanisms governing this device. However, it allows for a comprehensive qualitative understanding of the response while simultaneously reproducing the gross quantitative features present in the data.

When the Si is biased into accumulation, charge induced in the graphene is calculated by treating the junction as an ideal parallel-plate capacitor. Current through the graphene channel is then calculated in the standard manner^28^. Necessary graphene parameters—mobility and residual carrier concentration—were found by fitting device response when the device is optically saturated (see Supporting Information). Photogenerated carriers within the Si are assumed to recombine before gating the graphene when operating in accumulation.

When biased to deep depletion, the amount of photo-induced, dark and space charge collected in the potential well is calculated iteratively for a given time-step and gate-voltage until self-consistent convergence of space charge width, surface potential, and charge at the Si/ox interface is achieved (see Supporting Information). This charge is then balanced by varying the Fermi-level in the graphene by an amount corresponding to the interface charge density allowing for the photocurrent to be calculated. For all simulations, graphene is assumed to be an equipotential surface due to the low contact resistance (see Supporting Information) and the large magnitude of the back-gate voltage (typically in excess of 1 V) when compared to the channel bias voltage (100 mV). Charge loss within the gate dielectric (i.e., gate leakage) and any effects related to mobile charge within the oxide are ignored (see Supporting Information). Neglecting the potential drops across the contacts is valid owing to the small contact resistance compared to that of the graphene channel itself. With the use of other metals or a substantial increase in graphene mobility, the simplification would likely lose its veracity.

**Figure 4:**
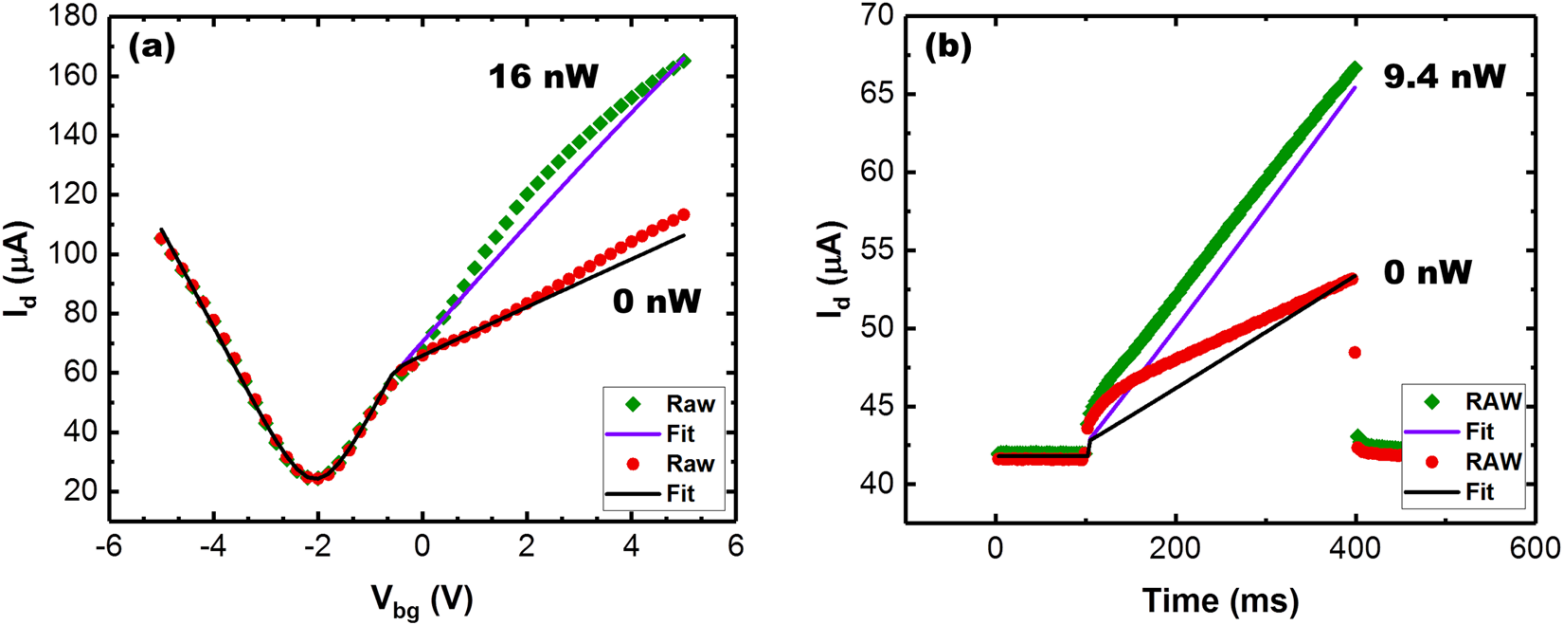
Comparison of experimental data with simulation for both dark and illuminated conditions as (**a**) V_bg_ is swept and (**b**) upon a sudden application of a constant back-gate voltage.

Figure 4a presents I_d_(V_bg_) data taken under dark and illuminated conditions compared to that predicted by the simulation as the back-gate is swept. For both cases, the I_d_(V_bg_) is well represented by the simulation in both accumulation and depletion. Transient behavior is also captured as shown by Fig. 4b, which plots the output of the GFET subject to a gate voltage pulse.

To first order, the simulation accurately predicts the response for the both the dark and illuminated conditions. There is, however, an offset between the actual and simulated curves. The largest offset is present in early transience for the dark response in Fig. 4b. We speculate that these offsets arise due to trapping and de-trapping of charge owing to non-idealities in the dielectric or defect-states at either the semiconductor/oxide or graphene/oxide interface. Regardless, the model—employing basic expressions for a MOS capacitor and a GFET—matches the behavior of the actual device closely and supports the interpretation of device operation. Importantly, the quantitative agreement between the model and data also allow for predictions of performance in improved devices, as well as indicating the necessary device parameters that should be improved for enhanced device functionality.

To this end, the operational limits of D^2^GOS-based detector were assessed as the present devices are limited both by the characteristics of the graphene channel and the Si/ox interface. For example, graphene mobility was measured to be 1,200 cm^2^/Vs despite published upper values for transferred CVD material exceeding 100,000 cm^2^/Vs^10^. Similarly, room temperature surface generation/recombination velocities can be reduced by at least 5x with further refinement of the HfO_2_ deposition technique^29^. Such improvements will enhance both responsivity and SNR.

**Figure 5:**
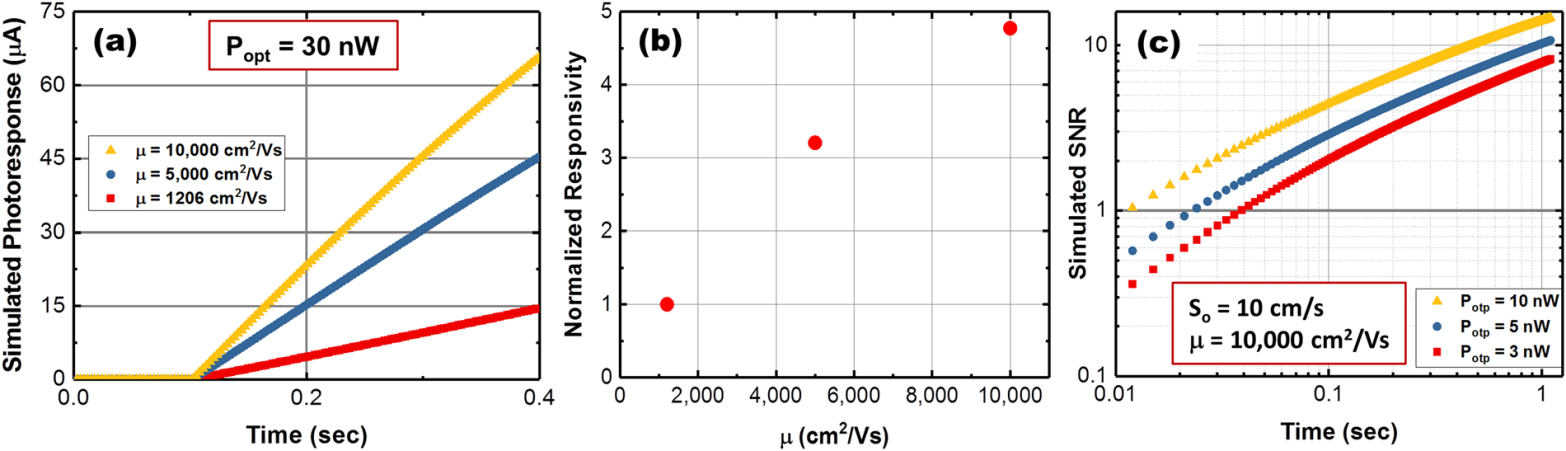
Prediction of device performance with increasing graphene mobility and reduced surface generation velocities (i.e, lower dark current). (**a**) Simulated photoresponse (I_d,Ligth_(t)–I_d,Dark_(t)) under pulsed back-gate measurement (V_bg_ = +3 V) for increasing mobility using a fixed illumination of 30 nW. (**b**) Simulated responsivities as a function of mobility extracted from I_d_(P_opt_) data (not shown) for different integration times (normalized to the responsivity attainable with commercial graphene presently available). (**c**) Simulated SNR for several low-level illumination powers using a surface generation rate of 10 cm/s and a mobility of 10,000 cm^2^/Vs.

Figure 5a plots the calculated time dependent photoresponse as the graphene mobility increases from 1,200 cm^2^/Vs (commercial film quality) to 10,000 cm^2^/Vs (high quality film). Improvement to SNR, by reducing the surface generation velocity from 480 to 10 cm/s, is also considered (see Supporting Information). Increasing mobility enhances the magnitude of the photoresponse (Fig. 5a) and thus the responsivity (Fig. 5b). Furthermore, a lessening of surface generation velocity reduces dark charge. Thus, substantial increases to SNR can be achieved for short integration times (Fig. 5c). Both the mobilities and the surface generation/recombination velocities used for these predictions have been previously realized^10,29,30^. Thus, the measured device performance reported here has the potential for significant improvement. Work is underway to this effect.

## Conclusion

We have presented the fabrication, characterization, and analysis of a hybrid graphene/depleted-absorber device termed here as a deeply-depleted graphene-semiconductor (D^2^GOS) detector. This scalable detector approach circumvents issues associated with absorbing light into atomically thin absorbing layers, as is the case for many graphene-based detectors. These devices, which have not been fully optimized, are capable of low-incident-power optical detection with responsivities exceeding 2700 A/W and dynamic range exceeding 30 dB at room temperature. Importantly, the device simultaneously integrates its signal even as it is continuously read-out opening up the possibility for alternative imaging modalities. This ability to integrate photogenerated signal combined with localized readout makes the D^2^GOS detector a self-sensing analog of a depleted MOS capacitor, which is the photo-charge collecting backbone of modern charge-coupled device technology. Additionally, the concept demonstrated here opens up the use of non-traditional semiconductors, such as indium arsenide (InAs), indium antimonide (InSb) and mercury cadmium telluride (HgCdTe/MCT), for use in charge integrating applications since the graphene removes the necessity of long-rang lateral charge transport and can be transferred atop virtually any substrate.

## Methods

### D^2^GOS Detector Fabrication

Devices were fabricated on 400 µm thick, n-type, low phosphorous doped <111> silicon wafers possessing a resistivity greater than 5000 Ωcm. The backside of the wafer was implanted with 75 As+ ions with an implant energy of 40 keV and an implant dose of 3 × 10^15^ ions/cm^2^ to form the back-side Ohmic contact. The implant was then activated by rapid thermal annealing (RTA) at 900 ^o^C for 30 s in a nitrogen atmosphere. Secondary ion mass spectroscopy (SIMS) revealed that the As was present at depths between 500–750 nm from the backside. The backside was then ion milled to remove any native oxide and a 20 nm/200 nm Ti/Au film was e-beam evaporated for the metal contact. The wafer was then rinsed in 1:4 BOE to remove any protective and native oxides and then ~50 nm of HfO_2_ was deposited by atomic layer deposition (ALD) at 250 ^o^C (TDMAH and H_2_O sources) onto the front side of the wafer for use as the gate dielectric. Graphene (Single Layer 1 cm × 1 cm Trivial Transfer Graphene- ACS Materials) was then transferred onto the substrate, rinsed in acetone, IPA, and then blown dry with nitrogen gas. The graphene detectors were then patterned by photolithography and etched by a 600 W O_2_ (gas flows and pressure) plasma in an asher for several minutes. Subsequently, 20 nm/200 nm Ti/Au etch stops were put down on the left and right side edges of the graphene detector using e-beam evaporation whereupon a passivation dielectric was deposited over the complete substrate. In order to ensure good adhesion of the dielectric onto the graphene, 1.5 nm of Al was first deposited by e-beam evaporation and then oxidized, followed by a 50 nm film of HfO_2_ by ALD. The etch stops were opened by a 65 s Cl_2_ etch through the HfO_2_ and the final Ti/Au source and drain contacts were patterned and deposited using e-beam evaporation.

### I_d_(V_bg_) Characterization

To characterize the I_d_(V_bg_) response of the graphene FET for both dark and illuminated conditions, a HP 4155B semiconductor parametric analyzer (SPA) was used. During a typical measurement, one of the SPAs source measurement units (SMU) supplied a small voltage of 100 mV to the device’s drain electrode (V_d_), while another SMU was used to hold the source electrode at ground (V_s_ = 0 V). A third SMU was used to sweep the V_bg_ (also referenced to ground) from an initial starting voltage to a final voltage in discrete V_bg_ steps. LabVIEW was utilized to record the source and drain currents as a function of V_bg_. Various voltage step times (SPA integrations times), ranging from 640 µs to 120 ms, were used during I_d_(V_bg_) sweeps to study the transient nature of charge integration in the D^2^GOS detector. All electronic measurements were made at room temperature in a Signatone probe station located inside an optically dark box that is shielded from EMI. To establish a connection with the back-gate electrode, devices rest on a conductive chuck located on the probe station.

### Pulsed Gate Electrical Characterization

To measure the temporal response of D^2^GOS elements a fast acquisition system was used. A Keithley 2400 applied a constant drain voltage and a Keithley 428 fast preamp was employed to measure the current flowing through the graphene channel. A National Instruments USB-6251 applied the back-gate voltage while monitoring the voltage output from the preamp, which is proportional to the input current. The USB-6251 has an analog output voltage slew rate of 20 V/µs, allowing for biasing of the Si into deep-depletion in under 1 µs. The acquisition was controlled using LabVIEW software.

### Optical Characterization

Photoresponse was characterized using laser sources—635 nm (Thorlabs S1FC635) and 405 nm (Thorlabs S1FC405)—that deliver light to the probe station’s optical microscope. Laser intensity was controlled below the system’s minimum output using neutral density filters. To account for losses by coupling into the probe station’s optics, the intensity of the laser light was measured after the objective lens using a power meter (Thorlabs S130VC) that was positioned on the probe station’s sample chuck. The output optical power at the objective was correlated to the laser controller’s power setting just prior to photo-response measurements. Beam size and shape were quantified using a DataRay Beam ‘R2 profiler. The beam was approximately Gaussian possessing a 1/e^2^ radius of 1690 µm. Measured responsivity and SNR presume the entirety of the measured power is absorbed despite the fact that the devices are a factor of 10 smaller. We take this step to account for the possibility of photogenerated holes, created away from the graphene channel, reaching the device and thus gating the graphene^17^.

## Electronic supplementary material

Supporting Information
