# Supplementary material for: Graphene-Insulator-Semiconductor Junction for Hybrid Photodetection Modalities
Source: Sci Rep. 2017 Nov 7;7:14651. doi: 10.1038/s41598-017-14934-4 (PMC5676778; doi:10.1038/s41598-017-14934-4)
Supplement: Supplementary file 1 — Supporting Information [file 41598_2017_14934_MOESM1_ESM.pdf]

Graphene-Insulator-Semiconductor Junction for Hybrid Photodetection Modalities

Stephen W. Howell*, Isaac Ruiz, Paul S. Davids, Richard K. Harrison, Sean W. Smith, Michael D. Goldflam, Nicholas J. Martinez, Jeffrey B. Martin, and Thomas E. Beechem

Sandia National Laboratories, Albuquerque, NM 87123

*Corresponding author: swhowel@sandia.gov

Supplemental information concerning the characterization and modelling of the deeply depleted graphene/oxide/semiconductor (D^2GOS) devices are provided below. Items are arranged in the order referred to in the main body of the text.

1) SIMS Characterization the Dopant Concentration

Secondary ion mass spectroscopy (SIMS) was utilized to quantify the dopant concentration of the silicon substrate on each side of the wafer. The n-type Si wafer (University Wafers) possessed a phosphorous dopant density of 10^{14} cm^{-3} on the side nearest to the graphene channel and was backside implanted with As at a density of 10^{19}-10^{20} cm^{-3} to realize an Ohmic body contact. Figure S1 provides the SIMS profiles acquired by Evans Analytical Group leading to these deductions.

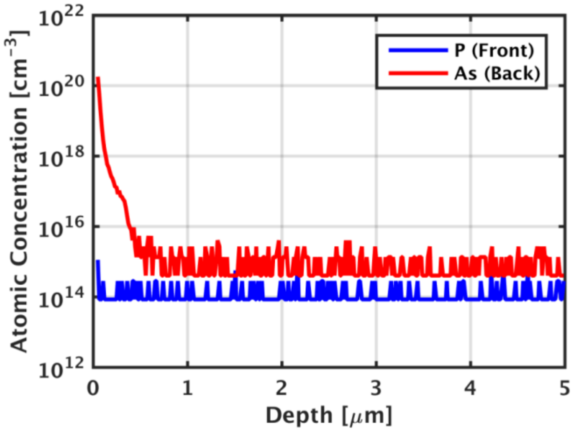

Figure S1. *Concentration of phosphorous and arsenic on the Si wafer's front surface and backside, respectively, as measured using SIMS.*

2) Back-Gate Sweep Rates Required for Deep Depletion

In a MOS capacitor, deep depletion occurs when the gate voltage changes rapidly enough that the device is unable to maintain thermal equilibrium (inversion charge does not form). The requisite rate of gate voltage change necessitating the onset of deep depletion can be estimated by[1]:

$$\frac{dV_{bg}}{dt} \gg \frac{qn_i}{2C_{ox}}\sqrt{\frac{\mu_{Si}V_t}{\tau_p}}, \tag{S1}$$

where C_{ox} is the oxide capacitance, μ_{Si} is the Si mobility, V_t is the thermal voltage (~ 26 mV), and τ_p is the minority hole lifetime. For low-doped Si used in this study (N_d = 9x10^{13} cm^{-3}), dV_{bg}/dt was found to be 0.8 V/s using an oxide thickness of 50 nm, a HfO_2 dielectric constant of 18.2, a Si mobility of 1404 cm^2/Vs,[2] and a minority carrier lifetime of 3x10^{-4} sec.

3) Electrical Characterization of GFET

To extract the electrical properties of the graphene FET (e.g., mobility and impurity concentration), the constant mobility model for transconductance is used[3,4]. The total resistance of a graphene channel (R_d) can be linked to the contact resistance and gate voltage using:

$$R_d = 2R_c + \frac{N_{sq}}{q\mu\sqrt{n_{imp}^2 + n_G\left(V_{bg}\right)^2}}, \tag{S2}$$

where μ is the electron mobility in the graphene channel, $n_G(V_{bg})$ is the carrier concentration due to capacitively coupled charge in the underlying gate material, n_{imp} is the residual carrier concentration on the graphene due to contamination and other sources,[5] N_{sq} is the number of squares, and R_c is the contact resistance of the graphene/metal interconnects. Equation S2 requires complete knowledge of the field-effect doping induced by the gate. Typically, degenerately doped Si is used to gate the graphene, which allows for straightforward calculation

via $n_G = C_{ox} V_{bg}$. During D^2GOS detector operation, a low-doped semiconductor operating in deep depletion is utilized instead. Determination of carrier density is therefore complicated due to the low dopant density inherent in deep depletion and the long time required to thermally generate inversion charge.

For these reasons, a high intensity light source is used to artificially invert the layer at the Si/ox interface during the $I_d(V_{bg})$ sweep. Under high intensity illumination, the potential well saturates with photo-induced hole charge. When saturation occurs, the amount of hole charge at the Si/ox interface can be approximated by $Q_{Si} \cong C_{ox} V_{bg}$ for $V_{bg} >> V_{fb}$, simplifying the calculation of $n_G(V_{bg})$.[6,7] Figure S2 shows a fit to data acquired during optical saturation using the GFET model (S2). From the fit, the graphene channel parameters are deduced to be: μ = 1206 cm^2/Vs, n_{imp} = 1.28×10^{12} cm^{-2}, and R_c ~ 35 Ohm. These values were found to be consistent across multiple devices.

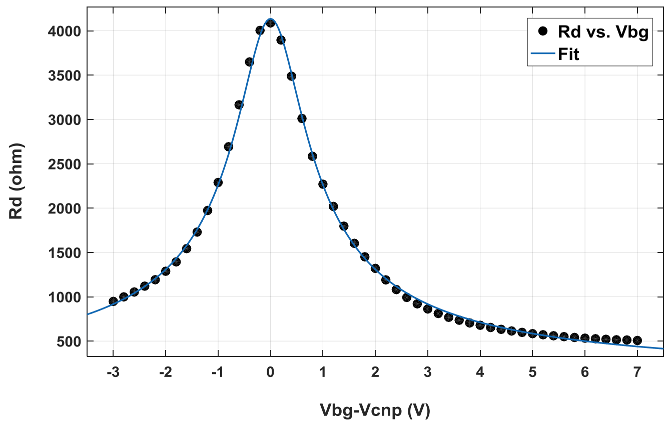

Figure S2. *Fit of measured resistance using the constant mobility model for a GFET. Resistance values are calculated from the saturated (optically induce inversion of the low-doped Si absorber) $I_d(V_{bg})$ data.*

4) CV Characterization of the Top-Gate Dielectric and Silicon Dopant Level

Capacitance-voltage (CV) measurements were used to determine the dielectric constant of the HfO_2 dielectric and to verify Si dopant density. Gold dot capacitors were incorporated near the D^2GOS detector arrays allowing for assessment of the HfO_2 and a determination of the

parameters required to deplete the underlying Si. To acquire CV data, a Hewlett-Packard 4284A was used in conjunction with a shielded probe station.

Figure S3 shows the measured capacitance and depletion depth (calculated from the capacitance data) for a dot capacitor (diameter =108 μm and oxide thickness of 50 nm). These data were taken using a drive frequency of 10 kHz. We estimate the HfO_2 dielectric constant to be ~ 18.2 by fitting a parallel-plate capacitor model to the capacitance values when the capacitor is biased into accumulation. From the capacitance values in depletion, we estimate the maximum depletion depth to be 2.7-3 μm. For the maximum depletion depth, we estimate the dopant concentration to be $N_d \sim 10^{14}$ cm^{-3}, which is close agreement with the SIMS data.

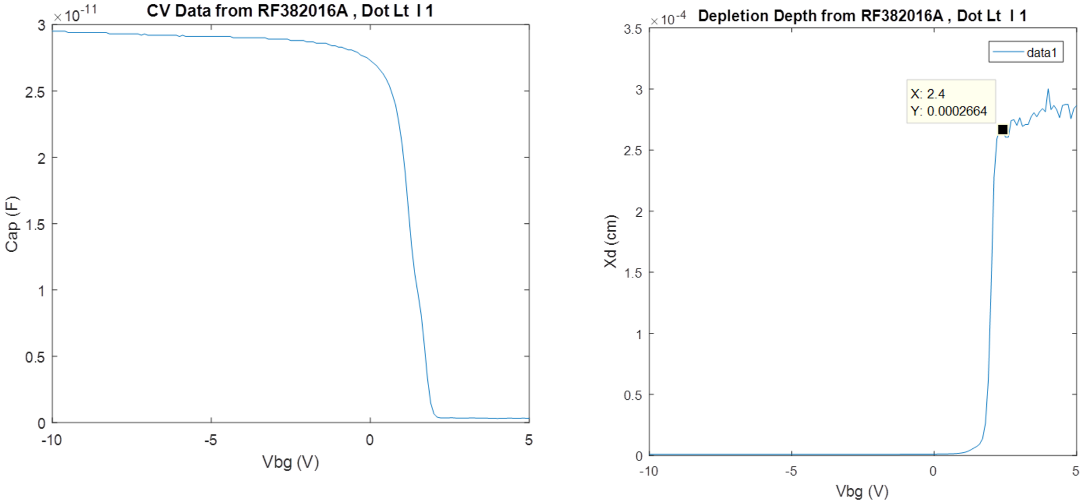

Figure S3. *(**a**) CV data from a 108 μm diameter dot capacitor located near the D^2GOS array. (**b**) Depletion depth as a function of back-gate voltage calculated for the capacitance data in (**a**).*

5) Extracting Time Dependent Responsivities for $I_d(V_{bg})$ and I_d(time) Data

Extracting Responsivities from $I_d(V_{bg})$ data

Figure 2a and b of the main text highlight not only variations with gate bias but also the time in which the substrate has been depleted since it takes longer to get from V_{fb} to higher voltages at a constant gate-voltage sweep rate. Qualitatively, more positive gate voltages induce higher currents in the graphene and larger responses to light. With larger V_{bg}, the potential well and depletion depth deepen increasing the well's storage capacity. Thus, more light can be both

absorbed and collected within the depletion region before saturation. Since V_{bg} is swept, the potential well's integration time also increases at larger gate voltages and allows for more hole collection. Increased hole collection results in larger electron density within the graphene and thus greater conductance.

Quantitatively, the responsivity (I_d as a function of optical power (P_{opt}) for a given V_{bg} and total potential well collection time) can be extracted from the $I_d(V_{bg},P_{opt})$ data in Fig. 2a of the main text. Since the step size (dV) and rate (t_{step}, SPA integration time) is constant during the sweep, the well's integration time (t_{Well}) corresponding to a particular back-gate voltage (V_{bg}) step can be estimated by marking the gate voltage where depletion begins (V_{fb}), knowing the number of steps required to obtain a particular back-gate voltage ($\frac{V_{bg}-V_{fb}}{dV}$), and the voltage step timing: $t_{Well}(V_{bg}) = \left(\frac{V_{bg}-V_{fb}}{dV}\right) * dt_{step}$. It should be noted that uncertainties associated with determining V_{fb} and dt_{step} will results in uncertainties in t_{Well}. Figure S4a shows I_d as a function of P_{opt}, for different V_{bg} values and estimated total potential well integration times. As mentioned above, since gate voltage is being systemically swept, larger V_{bg} values correspond to longer total collection times and a deeper potential well. For the case when V_{bg} = -1 V, there is little change in I_d as a function of P_{opt}, since the junction is in accumulation and there is no separation of electron-hole pairs. Traces, where the Si is biased into increasing depletion, show an increase in drain current for increasing optical illumination. The slope of each $I_d(P_{opt})$ curves also increases with increasing back-gate voltage, which is due to the increased potential well integration time and depletion depth. The ability to integrate photo-generated charge allows these devices to have responsivities in excess of 2,500 A/W using moderate integration times.

Extracting Responsivities from I_d(time) data

Figure S4b plots I_d as a function of intensity for several integration times from the data in Fig. 2b of the main text (101 ms, 200 ms, and 296 ms). These data show a linear response as the intensity increase from 0 nW to 9.4 nW. The offset in I_d, at 0 nW, is due to dark charge collecting in the potential well. Time dependent responsivities are determined by line fits to the $I_d(P_{opt})$ data.

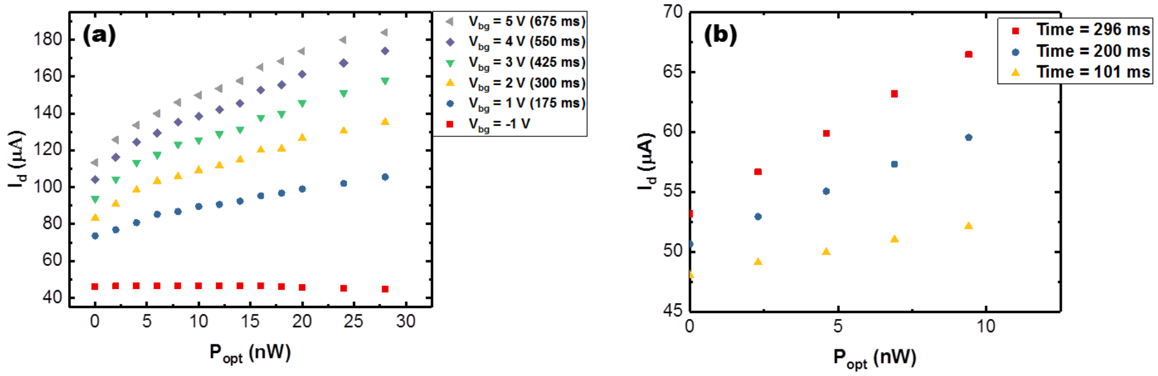

Figure S4. (**a**) I_d as a function of optical power for different V_{bg} values (extracted from Fig. 2a of the main text), which corresponds to different back-gate voltages and potential well integration times. (**b**) I_d response as a function of intensity for selected acquisition (well integration) times from Fig. 2b in the main text.

6) Additional D^2GOS Device Characterization with 635 nm Laser Source

Additional devices were characterized using a 635 nm laser light source beyond that reported in the main text. The devices were fabricated on two separate chips using the same commercial source of graphene and dielectric deposition tools as mentioned in the Methods section. Figure S5 shows $I_d(V_{bg})$ traces under dark and illuminated conditions for four separate D^2GOS elements. Quantitatively consistent behavior is observed for all elements investigated. The D^2GOS detector element discussed in the main text was located on chip-B.

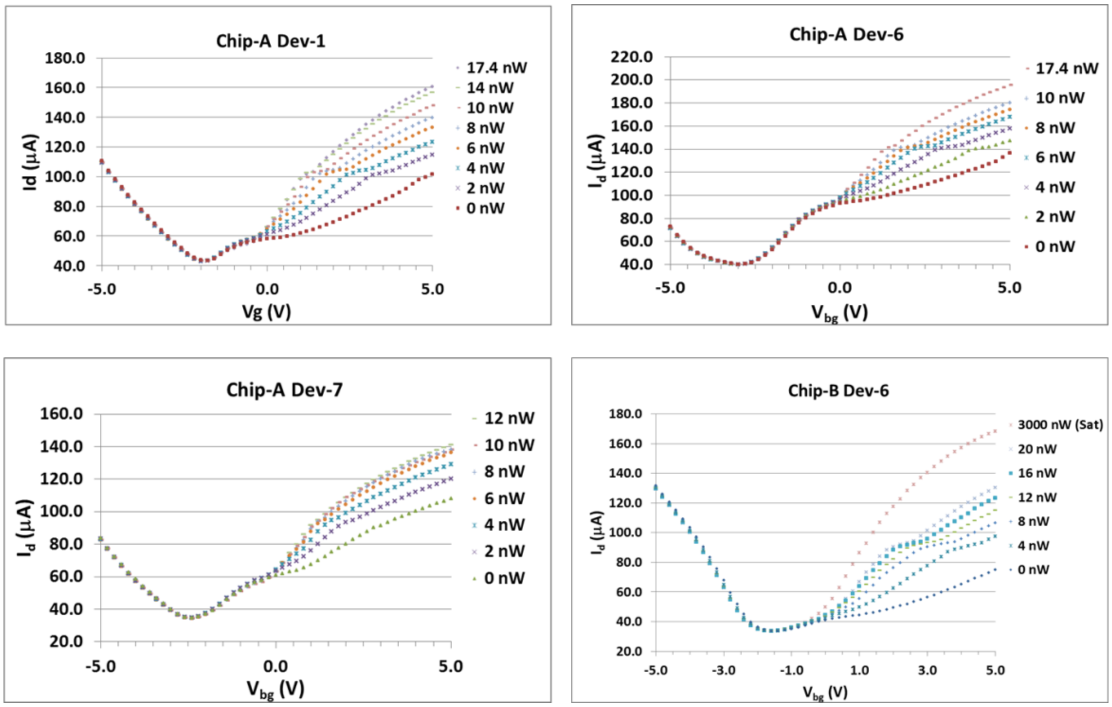

Figure S5. *Optical characterization using a 635 nm laser source for four additional D²GOS devices beyond that reported in the main text. Consistent behavior was observed for multiple devices on two different chips (A and B).*

7) D²GOS Detector Response under 405 nm Illumination

The optical response of several D²GOS devices was also characterized using a 405 nm laser light source. Figure S6 shows a typical D²GOS detector response for low-level illumination at 405 nm. Although the overall $I_d(V_{bg})$ behavior is similar, the measured responsivity is lower than when illuminated with a 635 nm laser source. The lower response is direct result of lower absorption into the Si layer for this wavelength as compared to 635 nm (see absorption simulation below).

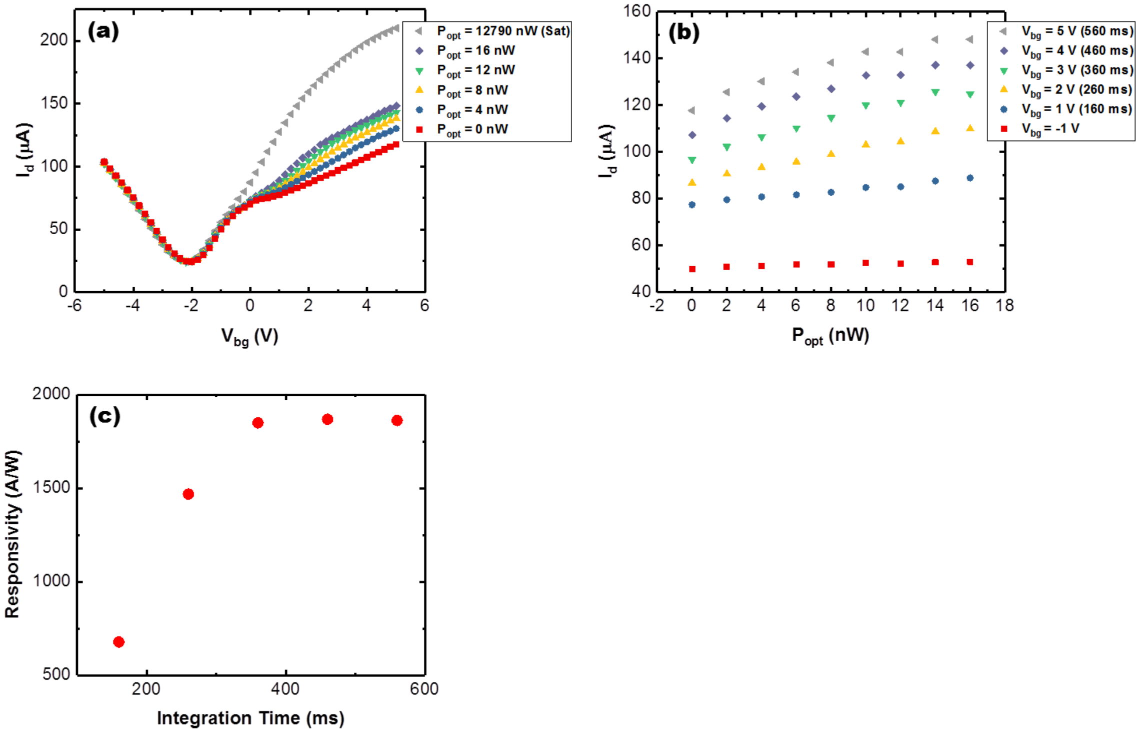

Figure S6. *Optical response of D²GOS detector under 405 nm illumination.* ***(a)*** *Current versus gate voltage at several optical powers along with resulting* ***(b)*** *intensity dependence and* ***(c)*** *responsivity.*

8) Repeatability and Lack of Hysteresis

Figure S7 presents consecutively acquired I_d(time) measurements where the back-gate was pulsed to 3 V for 300 ms (the time between 100 – 400 ms in the figure). During this measurement, there was a 200 ms delay between back-gate voltage pulses. Little hysteresis is observed between the acquired loops.

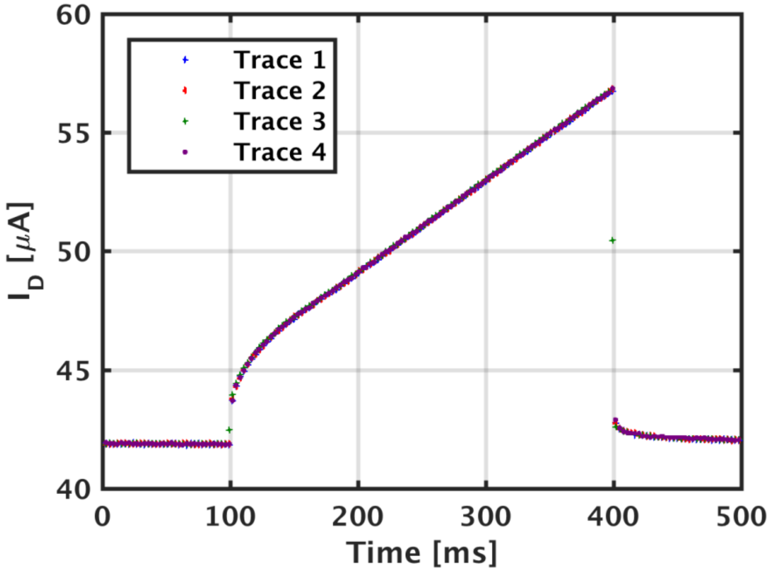

Figure S7. *I_d(time) data acquired for consecutive loops under the same test conditions. Very little change is observed for consecutive acquisitions of the pulsed V_{bg}.*

9) Dark Charge Well Saturation

Quality of the potential well forming beneath the Si/ox interface was gauged by monitoring the time required for I_d to saturate under dark conditions. Figure S8 shows drain current as a function of time for both dark and illuminated conditions. These data indicate that the potential well completely fills with dark charge in approximately 8 seconds, compared to a saturation time of ~ 1 second during an optical exposure of 22 nW (photo-induced charge).

The surface generation velocity (S_o) at the Si/ox interface can be estimated from the time required to saturate the well in Fig. S8. While other sources of dark charge emerge from silicon's properties (bulk generation/recombination, diffusion at edge of space charge region) and thus vary minimally with device processing, S_o is of particular interest owing to its sensitivity to process variations. Fitting the trace of Fig. S8 to the D^2GOS device model (see below), S_o is estimated at 400-1000 cm/s for our D^2GOS elements. Significant improvement from this value is anticipated with S_o values below 100 cm/s for HfO_2/Si interfaces having been reported[8].

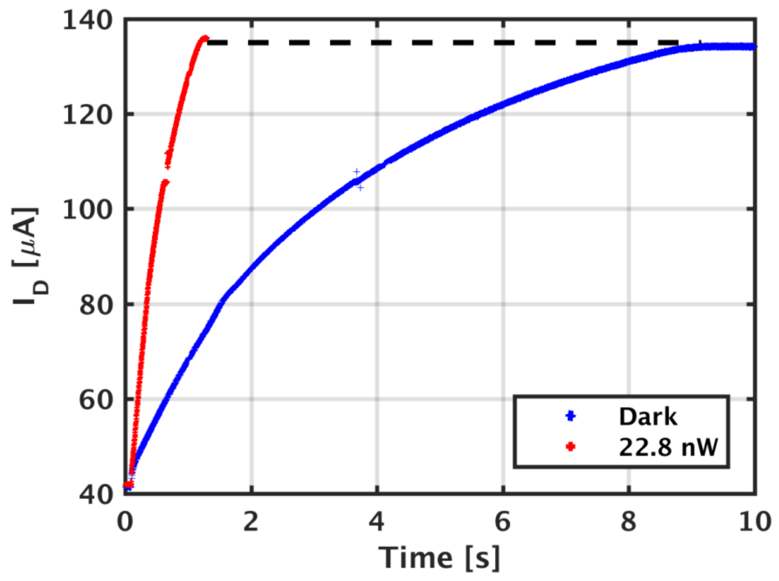

Figure S8. *Time required for the potential well of a* D^{2}GOS *element to saturate (saturation of I_d) due to dark charge generation and illumination. Well filling in the dark emerges primarily from surface generation and recombination at the Si/Ox interface.*

10) Quantifying Noise Sources

Noise was quantified by examining the dark current of D^{2}GOS devices under V_d and V_{bg} biases representative of operation. High frequency noise presumed to arise from thermal and shot sources was quantified at high frequencies (75-125 MHz) by assessing the variations in I_{ds} using a spectrum analyzer after saturation of the D^{2}GOS well[9]. Flicker, or 1/f noise, was quantified by acquiring the spectral noise density via analysis of the Fourier transform of the dark trace of Fig. S8.

Noise is probed during the filling of the well as this most closely mimics the device's operational conditions. This complicates analysis of the Fourier transform, however, as low frequency components account for the time-dependent increase in I_d associated with dark current filling the well rather than the noise of the system. To remove this artifact, the spectral noise density was quantified by analyzing the residual of a 3rd order polynomial fit over 1s increments of the dark trace of Fig. S8. The result is shown in Fig. S9. Results with this methodology were similar when analysis was performed when no potential well was present. High frequency results were

comparable between that acquired directly from the spectrum analyzer and from the Fourier transform. The normalized spectral noise density is typical of graphene devices[10].

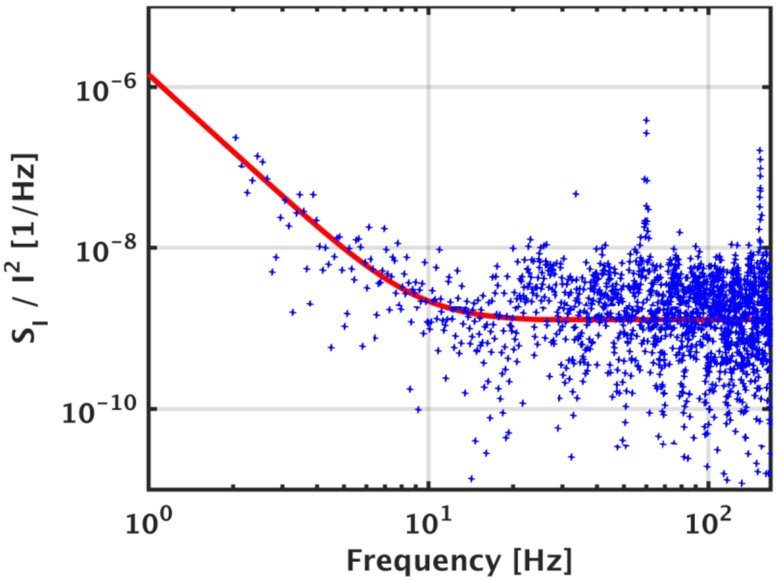

Figure S9. *Spectral noise density of* D^{2}GOS *devices. Values are comparable to previously reported graphene devices.*

11) Absorption into the D^{2}GOS Device Structure

Layer specific absorption of the D^{2}GOS device stack was quantified by solving Maxwell's equations via finite element analysis using COMSOL in the wavelength range of 200-800 nm. Published values of the optical constants of silicon[11] and HfO_2[12] were utilized while graphene's properties were modeled using the random phase approximation (RPA) with a carrier scattering time 60 fs.[13] This scattering time is in line with published values[14]. The response was independent to the Fermi-level of the graphene, which was varied from 0-1 eV. Graphene is modeled as a sheet of charge and its absorption quantified by integration of its losses via the relation

$$A_G = \frac{1}{P_{Inc}} \int \frac{1}{2} \sigma_1 \, |E|^2 da \,, \qquad \text{(S1)}$$

where P_{inc} is the incident power, σ_1 is the real part of graphene's conductivity, E the electric field at the graphene surface, and integration takes place over the surface. Results were verified by comparison to analytical calculations based on the transfer matrix method (TMM) where reflectance and absorption off the entire stack were nearly identical between the two methods. Figure S10 plots the absorption of the total device stack, as well as that absorbed within the silicon and graphene. For all wavelengths, graphene absorbs less than 2% of the incident light reaching the detector whereas the silicon absorbs 53% and 94% of the light at the examined wavelengths of 405 and 635 nm, respectively. Owing to the much greater absorption within silicon and the much longer source-drain spacing (200 μm) relative to graphene's recombination length (1 μm), photogating—as opposed to direct photoconduction—is therefore dominant.

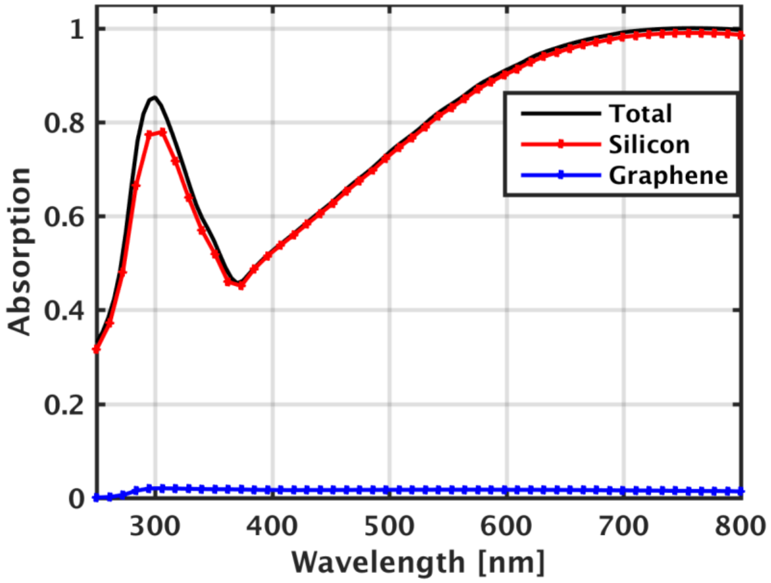

Figure S10. *Total absorption within the device stack along with that absorbed within the silicon and graphene layers. For all wavelengths, graphene absorbs less than 2%.*

12) Simulating D^2GOS Detector Operation

D^2GOS detector performance is qualitatively understood and quantitatively predicted using a one-dimensional semi-analytical model coupling "textbook" expressions governing the response of a metal-oxide-semiconductor (MOS) capacitor with a graphene field-effect transistor (GFET). Here, the simulation and the methodology of its employment are described. Practically, the

simulation first calculates the charge collected at the Si/ox interface and then quantifies how the current through the graphene changes owing to this electrostatically induced charge in the graphene arising from capacitive coupling.

To simulated $I_d(V_{bg})$ sweeps, an array of V_{bg} sweep values ($V_{bg,i}$) is first created based on the gate voltage step (dV_{bg}), starting gate voltage ($V_{bg,start}$), the final gate voltage ($V_{bg,end}$). The gate voltage parameters and the measurement step time (t_m) are selected to match the experimental conditions. For simplicity, the simulation handles conditions of accumulation and depletion separately. In accumulation, V_{bg} is below the flat-band voltage and graphene-oxide-semiconductor (GOS) junction acts like an ideal parallel-plate capacitor. Electron charge at the Si/ox interface is given by $Q_{Si}(V_{bg}) = C_{ox}(V_{bg}-V_{fb})$ for $V_{bg} < V_{fb}$, where $Q_{Si}(V_{bg})$ is the areal electron charge density in the Si at accumulation (C/cm^2) and C_{ox} is the capacitance of the gate oxide (F/cm^2). This charge will be balanced by the creation of hole carrier density ($n_{G\text{-}p}$) within the graphene channel via $n_{G\text{-}p}(V_{bg}) = |Q_{Si}(V_{bg})/q|$. Based on the discussion above, the GFET drain current (in accumulation) is calculated for every V_{bg} sweep value, where $V_{bg} \leq V_{fb}$, using the parallel plate model and equation S2 in conjunction with Ohm's law. Under accumulation, there is no build-up of photogenerated charge as shown in Fig. 2(a) of the main text. For this reason, photo-induced charge generation was set to zero in the simulation when the device operates in accumulation.

Under conditions of silicon depletion ($V_{bg} > V_{fb}$), electron density within the graphene channel ($n_{G\text{-}n}(V_{bg})$) is dictated by three interdependent parameters: (1) the back-gate voltage (V_{bg}), (2) the surface potential of the Si (ψ_S), and (3) the amount of time dependent charge residing in the Si/ox potential well (Q_{Well}). Q_{Well} is the summation of photo-induced holes (Q_{Sig}), holes generated from dark processes (Q_{Dark}), and positive charge from the trapped ionized dopants (Q_{SC}). The magnitude of Q_{Well} dictates the surface potential. The surface potential, in turn, determines the depletion depth and number of photo-induced holes among others. Owing to these interdependencies, an iterative, gate voltage and time-stepping approach is utilized.

For the initial V_{bg} sweep value ($V_{bg,0}$) immediately past the flat-band voltage (start of depletion), both dark and photogenerated charge has yet to enter the well. Thus, the initial amount of charge

in the potential well ($Q_{Well,0}$) can be set to 0 C allowing for the initial surface potential ($\psi_{S,0}$) to be calculated using[6]:

$$\psi_{S,0} = -V_{bg,0} - V_{fb} + \frac{Q_{Well,0}}{C_{ox}} - V_0 + \left[-2V_0\left(-V_{bg,0} - V_{fb} + \frac{Q_{Well,0}}{C_{ox}}\right) + V_0^2\right]^{\frac{1}{2}} \qquad \text{(S4)}$$

where $V_o = \frac{qN_d\varepsilon_o\varepsilon_S}{C_{ox}^2}$. Knowing $\psi_{S,0}$, the initial depletion depth ($W_{D,0}$) can be deduced via

$$W_{D,0} = \sqrt{\frac{2\psi_{S,0}\varepsilon_o\varepsilon_S}{qN_d}} \qquad \text{(S5)}$$

Possessing the depletion depth, the amount of optically-induced hole charge collected at a given measurement step time can be determined by[7]:

$$Q_{sig,0} = q\Phi\int_0^{t_m} \eta\, dt, \qquad \text{(S6)}$$

where Φ is the photon flux (photons/cm^2 s), and η is the quantum efficiency given by

$$\eta = 1 - \frac{e^{-\alpha W_{D,0}}}{1+\alpha L_p}, \qquad \text{(S7)}$$

where α is absorption coefficient of Si and L_p is the diffusion length for the minority hole carriers.

Absorbed flux is calculated via $\Phi = P_{Abs}^*/A_{Dev}$ where A_{Dev} is the area of the device and P_{Abs}^* is the amount of power absorbed in the device that is capable of gating the graphene. Experimentally, photons are incident on the surface from a beam that is, to first order, Gaussian possessing a 1/e^2 radius of 1690 μm (see Methods). Photoresponse can be quantitatively matched to experimental data assuming that photons absorbed within a 300 μm radius of the center of the device are capable of reaching the well and gating the graphene. This distance is within one diffusion length of the holes indicating that a purely one-dimensional treatment of the problem—while qualitatively reasonable—does not capture the entirety of the charge dynamics near the potential well.

This simulation also accounts for dark charge that originates from several sources for a MOS junction biased into depletion. The initial dark charge density ($J_{Dark,0}$) collecting in the potential well can be estimated by,[7]

$$J_{Dark,0} = \frac{qn_iW_{D,0}}{2\tau_p} + \frac{qn_iS_o}{2} + \frac{qn_i^2L_p}{N_d\tau_p}. \quad \text{(S8)}$$

where τ_p is the lifetime of the minority carrier terms and the terms (going left to right) correspond to: thermal generation of minority charge within the depletion zone, surface generation at the Si/ox interface, and diffusion of minority carriers generated within the bulk into the depletion zone. For a given measurement time step, the amount of dark charge can thus be calculated from $Q_{Dark,0} = \int_0^{t_m} J_{Dark,0}\, dt$. The space charge is calculated through $Q_{SC,0} = C'V_{bg,0}$ where C' is the junction capacitance under deep depletion given by:

$$C' = \frac{\epsilon_{ox}\epsilon_{Si}}{\epsilon_{Si}d + \epsilon_{ox}W_{D,0}} \quad \text{(S9)}$$

where ε_{Si} and ε_{ox} are the DC permittivity's of the Si and oxide, respectively. With the full charge within the potential well known, the initial electron carrier density in the graphene for the given $V_{bg,0}$ sweep value and time step is $n_{G\text{-}n,0}$= -($Q_{Sig,0}$ + $Q_{Dark,0}$ + $Q_{sc,0}$)/q. Knowing $n_{G\text{-}n,0}$ allows for Equation S2 to be utilized once again to deduce $I_{d,0}$.

For subsequent back-gate voltage steps, well charge from the previous step ($Q_{Well,i-1}$) is utilized to calculate the current surface potential ($\psi_{S,i}$). The surface potential is once again leveraged to calculate the proceeding depletion depth ($W_{D,i}$) allowing for signal ($Q_{Sig,i}$), dark ($Q_{Dark,i}$), and space charge ($Q_{sc,i}$) to be deduced ultimately providing $Q_{Well,i}$. This iterative process continues for every V_{bg}/dt step when the system is in depletion allowing for the quantification of photocurrent using equation S2.

The simulation methodology for $I_d(V_{bg})$ was also adapted to simulated I_d(time) measurements by calculating I_d while keeping the gate voltage fixed through each iterative step. Here the number of iterations is determined by the measurement step time and the total time the back-gate voltage

is applied (depleting the interface). Parameters used for these simulations are provided below in Table S1.

Table S1. *Parameters utilized to simulate photoresponse of* D^2GOS *devices.*

**Parameter**	**Symbol**	**Value**	**Note**
		**Oxide Properties**	
Oxide thickness	t_{ox}	50 nm	
Oxide dielectric const	ε_{ox}	18.2	Determined from CV
		**Silicon Properties**	
Dopant density	N_d	9e13 cm^{-3}	Determined from SIMS and CV
Hole lifetime	t_p	0.0003 sec	From Ref 2
Hole diffusion length	L_p	0.1 cm	From Ref 2
Surface generation vel	S_o	400-1000 cm/sec	
		**Graphene Properties**	
Graphene mobility	μ	1,206 cm^2/Vs	From GFET fit (saturated)
Impurity charge density	n_{imp}	1.3e12 cm^{-2}	From GFET fit (saturated)
Contact resistance	R_c	35 ohm	From GFET fit (saturated)
Offset charge (CNP)	$Q_{CNP-offset}$	5e-7 C/cm^2	Best fit
		**Optical Properties**	
Beam FWHM		0.2 cm	From beam profile measurement
Wavelength	λ	635 nm & 405 nm	Laser sources
Si absorption coefficient	α	3774.27 cm^{-1} & 102031.9 cm^{-1}	

Since graphene devices have demonstrated operational speeds > 100 GHz, the response time will be primarily limited by how fast photogenerated minority carriers collect in the potential well at the absorber/oxide interface. This collection time depends both on how long it takes minority carriers to diffuse into the depletion zone from the bulk (minority diffusion time), as well as how fast electron/hole pairs are separated in the depletion region. This separation time depends on both the mobility of minority carriers in the absorber and the magnitude of the internal electric field near the absorber/oxide interface. Using absorbers with high minority mobility and fast diffusion time will increase operational speed. In addition to the minority carrier dynamics, the device capacitance will also impact response time.

13) Raman Characterization

Raman linescans were performed along the midplane of the horizontal and vertical axes of devices using a WiTec alpha300R system with a 100X/0.95 NA objective and 1 mW of 532 nm incident light. The Raman scattered light was dispersed using a WiTec UHTS visible

spectrometer with a 600 l/mm grating resulting in spectral accuracy of +/- 1 cm^{-1}. Spectra were acquired every 333 nm with minimal variation observed across the devices. Figure S10 shows a representative Raman spectrum from D^2GOS device active area. From the spectrum, three characteristics are of note. First, the Raman spectrum is consistent with monolayer graphene of minimal disorder as evidenced by the lack of signal in the region near 1350 cm^{-1} indicative of defects. Second, the slight background present on the spectrum is most likely due to residual contaminants remaining from fabrication processing. Finally, fitting of the peak positions indicates a residual doping on the order of 10^{12} cm^{-3} consistent with fitting from the GFET response under saturation[15].

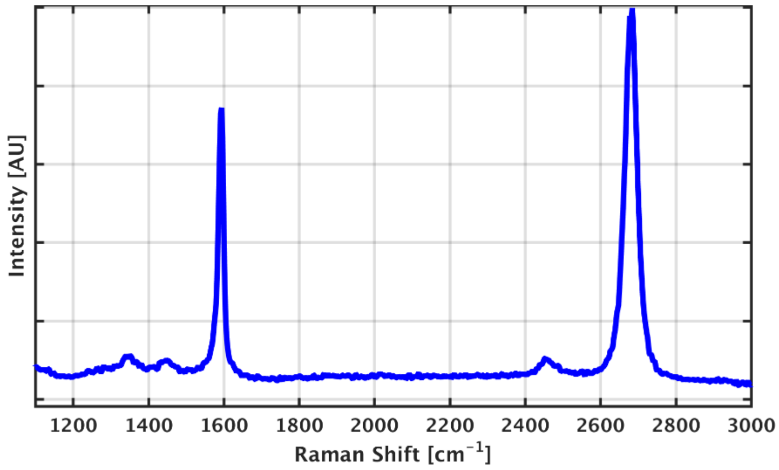

Figure S11. *Representative Raman spectrum of* D^2GOS *detector indicating monolayer of comparatively small disorder and residual doping on the order of* 10^{12} cm^{-3}.

12) Simulating the Impact of Surface Generation on SNR

To gauge the impact of surface generation, SNRs are simulated for improved surface generation velocities (S_o). Figure S12a-c shows simulated I_d traces under dark (0 nW) and illuminated (10 nW) conditions for various values of S_o (480 cm/s, 100 cm/s, and 10 cm/s). For the illuminated case, the simulated I_d is a combination of photo-generated charge and dark charge (caused by the dark charge generation processes discussed above). The I_d simulations shown in Fig. S12 clearly demonstrate the impact of reducing S_o. Figure S12d, shows a simulated SNR (using equation 1 from the main text). By suppressing S_o to manageable values (10 cm/s has been recently

demonstration for a HfO_2/Si interface), the SNR approaches the photon shot noise limit for moderate integrations times (ideal for 10 nW illumination).

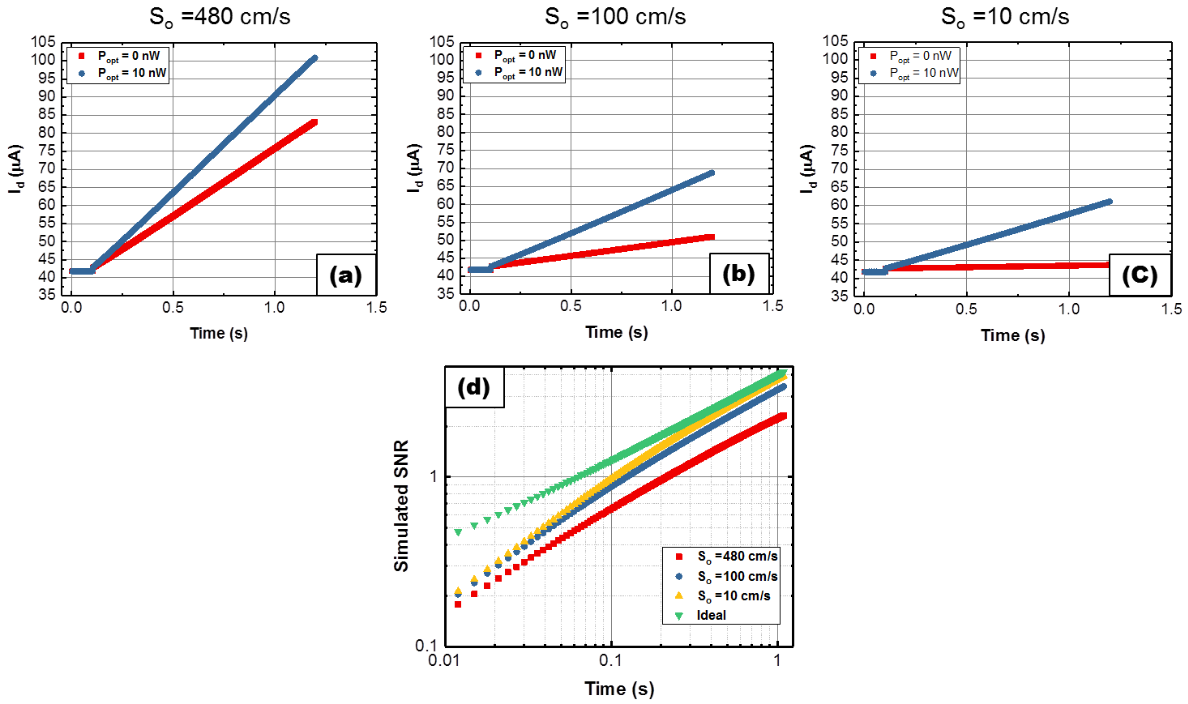

Figure S12. *I_d simulations for different surface generation rate.* ***(a)*** *S_o = 480 cm/s as measured from the device data,* ***(b)*** *S_o = 100 cm/s, and (c) S_o = 10 cm/s (a value that has been obtained experimentally).* ***(d)*** *SNR, for different $S_{o,}$ calculated using the simulated I_d signal. For these simulations, V_d = 100 mV and the graphene mobility is 1206 cm^2/Vs.*

References

1. Van Zeghbroeck, B. Principles of Semiconductor Devices. http://ecee.colorado.edu/~bart/book/.
2. Semiconductors on NSM. http://www.ioffe.ru/SVA/NSM/Semicond/index.html.
3. Kim, S.; Nah, J.; Jo, I.; Shahrjerdi, D.; Colombo, L.; Yao, Z.; Tutuc, E.; Banerjee, S. K., Realization of a High Mobility Dual-Gated Graphene Field-Effect Transistor with Al_2O_3 Dielectric. *Appl. Phys. Lett.* **2009,** *94* (6), 062107.
4. Venugopal, A.; Chan, J.; Li, X.; Magnuson, C. W.; Kirk, W. P.; Colombo, L.; Ruoff, R. S.; Vogel, E. M., Effective Mobility of Single-Layer Graphene Transistors as a Function of Channel Dimensions. *J. Appl. Phys.* **2011,** *109* (10), 104511.

5. Dorgan, V. E.; Bae, M.-H.; Pop, E., Mobility and Saturation Velocity in Graphene on SiO_2. *Appl. Phys. Lett.* **2010,** *97* (8), 082112-082112-3.
6. Rogalski, A.; Adamiec, K.; Rutkowskit, J., *Narrow-Gap Semiconductor Photodiodes*. SPIE-The International Society for Optical Engineering: Bellingham, Washington, 2000.
7. Sze, S. M.; Ng, K. K., *Physics of Semiconductor Devices: Edition 3*. John Wiley & Sons: 2006.
8. Repo, P. Reducing Surface Recombination in Black Silicon Photovoltaic Devices using Atomic Layer Deposition. 2016.
9. Kayyalha, M.; Chen, Y. P., Observation of Reduced 1/f Noise in Graphene Field Effect Transistors on Boron Nitride Substrates. *Appl. Phys. Lett.* **2015,** *107* (11), 113101.
10. Balandin, A. A., Low-Frequency 1/f Noise in Graphene Devices. *Nat. Nanotechnol.* **2013,** *8* (8), 549-555.
11. Green, M. A.; Keevers, M. J., Optical Properties of Intrinsic Silicon at 300 K. *Progress in Photovoltaics: Research and Applications* **1995,** *3* (3), 189-192.
12. Materials Database. http://sspectra.com/sopra.html.
13. Falkovsky, L. In *Optical Properties of Graphene*, Journal of Physics: Conference Series, IOP Publishing: 2008; p 012004.
14. Yan, H.; Li, X.; Chandra, B.; Tulevski, G.; Wu, Y.; Freitag, M.; Zhu, W.; Avouris, P.; Xia, F., Tunable Infrared Plasmonic Devices using Graphene/Insulator Stacks. *Nat. Nanotechnol.* **2012,** *7* (5), 330-334.
15. Schmidt, D. A.; Ohta, T.; Beechem, T. E., Strain and Charge Carrier Coupling in Epitaxial Graphene. *Phys. Rev. B* **2011,** *84* (23), 235422.
